# Cross-Reactivity of Antiphospholipid Antibodies with Gut Commensal Proteins in Antiphospholipid Syndrome

**DOI:** 10.1055/a-2868-5248

**Published:** 2026-06-03

**Authors:** Dagmar J.M. van Mourik, Manon Balvers, Valérie L.B.I. Jansen, Patrick A. de Jonge, Michiel Coppens, Max Nieuwdorp, Saskia Middeldorp, Jeroen C.J. Eikenboom, Jan Voorberg, Thijs E. van Mens

**Affiliations:** 1 Division of Thrombosis and HemostasisDepartment of Internal Medicine 4501 Leiden University Medical CenterLeidenSouth Hollandthe Netherlands; 2 Einthoven Laboratory for Vascular and Regenerative Medicine 4501 Leiden University Medical CenterLeidenSouth Hollandthe Netherlands; 3 Department of Vascular Medicine 26066 Amsterdam UMC, Location University of AmsterdamAmsterdamNorth Hollandthe Netherlands; 4 Amsterdam Cardiovascular Sciences, Pulmonary Hypertension and ThrombosisAmsterdamNorth Hollandthe Netherlands; 5 Amsterdam Reproduction and DevelopmentAmsterdamNorth Hollandthe Netherlands; 6 Amsterdam Gastroenterology, Endocrinology and Metabolism; Endocrinology, Metabolism and Nutrition, Amsterdam UMCAmsterdamNorth Hollandthe Netherlands; 7 Department of Internal Medicine 569645 Radboud University Medical CenterNijmegenGelderlandthe Netherlands; 8 159217 Sanquin ResearchAmsterdamNorth Hollandthe Netherlands

**Keywords:** antiphospholipid syndrome, autoimmune diseases, cross-reactivity, intestinal microbiome, molecular mimicry

## Abstract

**Background:**

Antiphospholipid syndrome (APS) is an autoimmune disease characterized by the persistent presence of antiphospholipid antibodies (aPL), mainly targeted against β2 glycoprotein 1 (β2GP1). The autoimmune response to β2GP1 is aimed at several B-cell and T-cell epitopes. Molecular mimicry of these epitopes by gut commensal proteins, so-called mimotopes, causing cross-immunization, might contribute to the formation of aPL.

**Objective:**

To study the potential role of gut microbiome cross-immunization in APS by examining cross-reactivity of aPL with gut commensal mimotope-containing proteins.

**Methods:**

Fecal microbial metagenome of APS patients was determined using shotgun sequencing. An in-house developed in silico pipeline was used to identify gut commensal proteins that show sequence homology with known β2GP1 B and T cell epitopes in the metagenomic data. An enzyme-linked immunosorbent assay was used to test the identified microbial proteins for IgG cross-reactivity, with plasma of 21 APS patients and 17 control participants.

**Results:**

The in silico pipeline resulted in the identification of six gut commensals with a B cell and T cell β2GP1 epitope homologue. Of these, YjjG family noncanonical pyrimidine nucleotidase, one of the candidate-β2GP1 B cell mimicking proteins, showed significantly increased IgG reactivity in APS patients compared to control participants, as well as higher binding of a specific anti-β2GP1 monoclonal antibody than a negative control.

**Conclusion:**

Our study shows reactivity of IgG antibodies to YjjG family noncanonical pyrimidine nucleotidase from *Roseburia amylophila* in APS patients. Insights into the origins of antibody formation may yield new therapeutic targets for improvement of APS treatment.

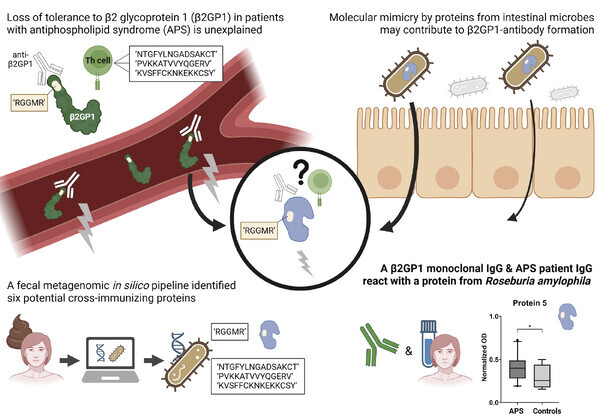

## Introduction

The antiphospholipid syndrome (APS) is a devastating autoimmune disease, characterized clinically by thrombosis and pregnancy morbidity. 1 APS patients are amongst the most difficult to treat thrombosis patient groups, with a high prevalence of recurrent thrombotic events despite anticoagulant treatment. The efficacy of secondary prevention of pregnancy complications with antithrombotic drugs is also limited. Lack of understanding of the origin of autoimmune antibodies in APS hinders the development of curative treatments.

Accruing evidence implicates a role of the intestinal microbiome in the development of autoimmune diseases. 2 For instance, systemic translocation of the intestinal microbe Enterococcus gallinarum promotes autoimmunity in systemic lupus erythematosus (SLE), a disease that greatly overlaps with APS. 3 We have recently shown that altering the gut microbiome also affects indicators of APS disease activity, providing proof of concept of a causal role for intestinal gut microbiome in human APS. 4 The mechanism, however, remains to be established, as studies into microbiome composition so far do not show convincing differences between APS patients and controls, 5 6 nor did we find evidence for a role of increased intestinal permeability in human APS. 4 5

APS is serologically defined by the presence of antiphospholipid antibodies (aPL), a group of autoantibodies that are either directed at cell membrane phospholipids or cell membrane phospholipid-bound proteins. aPL are persistently present in APS but are also known to transiently occur in response to infections. Molecular mimicry, that is, sequence homology between self- and nonself-peptides, is a plausible explanation for this phenomenon and is observed in other autoimmune diseases. 7 Specific aPL-triggering pathogens with peptide homology to APS autoantigens have indeed been identified. 8 Peptides homologous to epitopes are referred to as mimotopes (mimicking epitopes).

In contrast to infectious triggers, the intestinal microbiome represents a permanent source of potential cross-immunizing antigens. In support of this hypothesis, molecular mimicry of a cardiac autoantigen by a peptide expressed by gut commensal *Bacteroides thetaiotaomicron* elicits an autoimmune response in a myocarditis mouse model, and patient antibodies and T cells cross-react with the microbe and its peptide. 9 Similarly, human Ro60 mimicking gut microbial peptides have been identified that cross-react with anti-Ro60 antibodies and T cells from SLE patients, and colonization of germ-free mice with these microbes led to the development of autoimmunity. 10

Cross-immunization triggered by gut microbial antigens may also contribute to APS pathogenesis. The plasma protein β2 glycoprotein 1 (β2GP1) contains the major APS B cell autoepitope, p39-43 (RGGMR). 11 The human gut commensal *Roseburia intestinalis* expresses DNA methyltransferase (DNMT) that contains an exactly matching sequence to the B cell autoepitope, as well as another protein that contains a sequence homologous to a known APS T cell autoepitope. 6 In a series of experiments, it was shown that APS patient-derived antibodies and T cells cross-react with *R. intestinalis* and its mimicking peptides; anti-β2GP1 titers correlate with anti-DNMT titers in patient plasma; mice immunized with *R. intestinalis* develop anti-β2GP1 antibodies; and oral administration of *R. intestinalis* to autoimmune-prone mice leads to the formation of aPL and APS phenotype. 6

In line with these findings, β2GP1 mimicking proteins from the intestinal microbiome may contribute to APS pathophysiology through cross-immunization. The aforementioned work identified a homolog to a single T cell epitope in β2GP1. 6 In fact, there are up to six different β2GP1 epitopes described in the literature, corresponding to different HLA types, that induce a T cell response in APS patients. 12 13 14 15 For these T cell autoepitopes, it is biologically equally plausible that the intestinal microbiome contains mimicking peptides. We therefore aimed to evaluate the presence of gut microbial proteins mimicking β2GP1 epitopes in APS patients, using a purpose-built bioinformatic pipeline based on an expanded number of β2GP1 T cell epitopes, with input from patients' microbiome sequence data, and test these in silico identified intestinal microbial proteins for immunoglobulin reactivity using plasma of APS patients.

## Methods

### Study Population

Thrombotic and/or obstetric APS patients diagnosed according to the 2006 Sydney classification criteria were included from the Amsterdam APS biobank (Ethical Review Board number 2015_347). 1 Additionally, for this study on β2GP1 cross-reactivity, subjects had to have a positive result of their latest anti-β2GP1 IgG or IgM test, with the clinically applied 99th percentile as a cut-off. Since the vast majority of eligible patients were female, we chose to exclude males for homogeneity. Further exclusion criteria comprised microbiome affecting factors (i.e., gastro-enteritis, chronic intestinal diseases, use of antibiotics, or protein pump inhibitors in the last 3 months). Patients were recruited at the outpatient clinic of our tertiary care vascular medicine department. Control participants were recruited through the included patients, from their social environment, to ensure comparability of the source population in terms of microbiome composition, excluding family or household members to exclude co-housing and genetic effects. The same in- and exclusion criteria applied, aside from the APS-related criteria. We collected feces and EDTA plasma. All participants provided written informed consent. The Amsterdam UMC Biobank Committee approved the study, and the study was conducted in agreement with the Declaration of Helsinki.

### Identification of Intestinal Microbiome

Feces samples of APS patients and control participants were collected at home no longer than 24 hours preceding the study visit. Samples were stored at 4°C until study visit and stored at −80°C until processing. A previously described bead-beating method was used for fecal DNA isolation. 16 Shotgun sequencing of the isolated DNA was obtained by Illumina Novaseq 6000 sequencing with 2 × 150 bp paired-end reads (Novogene, Cambridge, United Kingdom).

### β2GP1 Epitope Selection

We selected p39-43 (RGGMR) as B cell epitope, which is considered to be the immunodominant B cell epitope in β2GP1. 11 There are up to six T cell epitopes present in β2GP1. 12 13 14 15 We selected three HLA-restricted T cell epitopes, covering different parts of β2GP1: p92-105 (NTGFYLNGADSAKCT) on domain II, p248-261 (PVKKATVVYQGERV) on domain V, and p276-290 (KVSFFCKNKEKKCSY) on domain V. 13 14 We eliminated the epitope present in the signal peptide. 12 For the three strongly overlapping epitopes present between p246-261 in β2GP1, we selected one to represent this area.

### In Silico Pipeline for Microbial Protein Selection

**Fig. 1:**
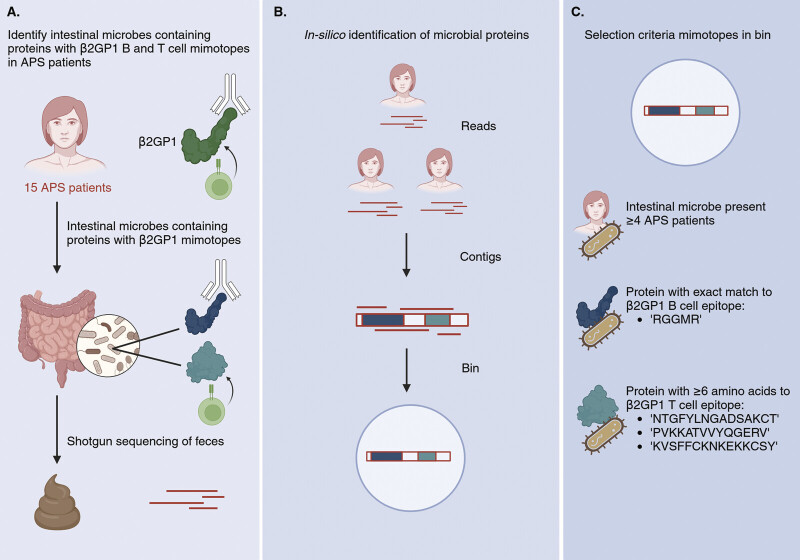
In silico pipeline method. The in silico pipeline was used to identify intestinal microbes containing proteins with β2GP1 B and T cell mimotopes. Shotgun sequencing was used to identify the genomes of the present intestinal microbes ( **A** ). Overlapping DNA reads from the shotgun sequencing were concatenated into contigs; similar contigs were binned together to form a metagenome ( **B** ). The metagenomes were used to identify proteins with β2GP1 mimotopes. Selection criteria consisted of the presence of the protein combination in at least four APS patients, a protein with an exact match to the B cell epitope, and a protein with at least six consecutive amino acids aligned to one of the T cell epitopes in the same organism ( **C** ). (Created in BioRender. van Mourik, D. [2025] https://BioRender.com/h67e259 ).

An in silico pipeline was used to identify gut microbial proteins with an epitope homologous to the β2GP1 B cell autoepitope, with the requirement that an epitope homologous to a T cell autoepitope was also present in the same metagenome in APS patients ( Fig. 1 ). The detailed in silico pipeline is described in Supplementary Materials . In short, the approach started with fecal shotgun sequencing data of APS patients as a source to identify the presence of β2GP1-homologous gut microbial proteins. Processing of the sequencing data started with a standard quality check, followed by the selection of nonhuman reads. Per sample, nonhuman reads were assembled into contigs, which are overlapping DNA segments. Assemblies of all samples were concatenated to form an overall assembly, and the contigs of the cleaned overall assembly were binned into larger metagenome-assembled genomes. Prediction of protein-coding genes on the overall cleaned assembly was performed by Prodigal (v2.6.3). 17 The protein sequences were converted to a protein database, which was queried for homologous segments with the T cell epitopes of interest, and only ungapped alignments were considered. Since the B cell epitope was small, five amino acids, only exact matches were selected. Identified T and B cell mimotopes were assigned to a binned metagenome. Mimotopes can be present on different contigs but originate from the same protein; mimotopes from the same protein were clustered. Clustered T and B cell mimotope combinations in the same bin were assigned to a unique protein cluster. Protein clusters present in at least four APS patients were considered; further quality checks included 50% coverage of both full proteins and a combined average read depth of at least 1. Protein-to-protein BLAST in the NIH protein database was used to identify the protein names and subsequent species of eligible protein clusters. The Swiss-Model was used to predict the tertiary structures of B-cell mimotope-containing proteins for accessibility of antibody-binding. Finally, proteins were selected for in vitro testing when they met these criteria.

#### Microbial Protein Production

The BLAST sequences of the complete B-cell mimicking microbial proteins were used for protein production at Genscript, New Jersey, United States. Genscript used *Escherichia coli* for protein expression and used a nickel column for purification. Concentrations were determined by Bradford, purity was verified in SDS-PAGE, and the proteins were confirmed using LC-MS. All proteins had a purity of ≥ 90%.

### Enzyme-Linked Immunosorbent Assay

As the primary outcome, we assessed identified gut microbial proteins for IgG reactivity in APS patients and control participants. Secondary outcomes consisted of anti-β2GP1 IgG monoclonal reactivity and APS plasma IgA and IgM reactivity to the gut microbial proteins. An in-house developed enzyme-linked immunosorbent assay (ELISA) was used to assess plasma-IgG reactivity to the identified B-cell mimicking microbial proteins. Corning plates (Corning 96-well EIA/RIA Easy Wash Clear Flat Bottom Polystyrene High Bind Microplate) were coated with 5 μg/mL plasma-purified β2GP1 (B2GP1-0001 from Prolytix, formerly Haematologic Technologies), human recombinant insulin (Tocris Bioscience) as a negative control, or identified proteins in Tris buffer, pH 7.6, and incubated overnight at 4°C. Block buffer (Pierce Protein-Free Blocking Buffer) was incubated for 1 hour at room temperature. EDTA plasma of the participants was diluted 1:100 using PBS with 0.1% Tween-20 and incubated with low shaking at room temperature for 2 hours. The secondary antibody was diluted according to the manual for IgG 1:20,000 (ThermoFisher, Goat anti-Human IgG [H + L] Secondary Antibody, HRP) and incubated at room temperature for 30 minutes. Plates were developed using o-phenylenediamine dihydrochloride and stopped by 2 M sulfuric acid. The Byonoy Absorbance 96 was used to measure the absorption at 490 nm. A reference curve was produced using 1:50 to 1:2,000 diluted plasma of an independent APS patient, with a history of pregnancy morbidity (premature delivery due to HELLP and preeclampsia in two pregnancies) and serological positivity for anti-β2GP1 IgG, anti-cardiolipin IgG and IgM, and lupus anticoagulant. The titer of anti-β2GP1 IgG was 70.8 U/mL. The curve was used to confirm technically adequate measurement and linearity at the chosen reference dilution of 1:100. All other measured optical densities were normalized to this reference plasma dilution. The main read-out was normalized OD, defined as optical density (average of sample plasma in triplo at 1:100)/optical density (average of reference plasma in triplo at 1:100). Secondary outcomes IgA and IgM responses were tested similarly with respective secondary antibodies (IgA 1:20,000, ThermoFisher, Goat anti-Human IgA Secondary Antibody, HRP; or IgM 1:10,000, ThermoFisher, Goat anti-Human IgM Secondary Antibody, HRP), with optical densities normalized to 1:100. Secondary antibodies were not subclass specific. A specific β2GP1 mouse monoclonal antibody against domain I, 3B7 (4.1 mg/mL 18 ), was additionally tested in the ELISA to assess the specificity of the proteins of interest. Previous studies showed the formation of β2GP1-3B7 complexes, confirming 3B7 as an anti-β2GP1 monoclonal antibody. 19 20 Although epitope-mapping has not been performed, it is proposed that the affinity of 3B7 for domain I of β2GP1 is likely higher compared to other domains because the monoclonal does show activity in the lupus anticoagulant assay. 18

### Analysis

Descriptive statistics were analyzed with SPSS version 29.0 and used to summarize baseline characteristics. We used an independent *t* -test for BMI and age, and a Fisher's exact test for statistical analysis of the baseline characteristics in Table 1 . A comparison of APS patients and control participants was made for all ELISA outcomes. Normalized ODs were used for analysis. The Mann–Whitney U test was used to analyze the ELISA data, and Spearman's rho was used for correlation analysis of the ELISA data. *p* -values below 0.05 were considered statistically significant. ELISA data were analyzed and visualized in GraphPad Prism version 9.3.1.

**Table 1:** Baseline characteristics

	APS patients ( *n* = 21)	Control participants ( *n* = 17)	*p* -Value
Age, mean (± SD)	35.9 (8.6)	41.5 (12.7)	0.116
Female, *n* (%)	21 (100)	17 (100)	
BMI, mean (± SD)	24.2 (4.0)	24.5 (4.1)	0.848
Current smoking, *n* (%)	3 (14.3)	3 (17.6)	1.000
SLE, *n* (%)	3/20 (15.0)	0 (0.0)	0.238
History of thrombosis, *n* (%)			
Venous thromboembolism	7 (33.3)	1 (5.9)	0.053
Arterial thromboembolism	9 (42.9)	0 (0.0)	0.002
History of pregnancy, *n* (%)	15 (71.4)	14 (82.4)	0.476
History of obstetric events, *n* (%)			
Late fetal death	10/15 (60.0)	2/14 (14.3)	0.008
≥3 early losses	2/15 (13.3)	0 (0.0)	0.483
Preterm birth	2/15 (13.3)	0 (0.0)	0.483
Obstetric APS a , *n* (%)	11 (52.4)	NA	
Thrombotic APS a , *n* (%)	16 (76.2)	NA	
Antiphospholipid antibodies, *n* (%)			
Anti-β2GP1 IgG	18 (85.7)	Not tested	
Anti-β2GP1 IgM	5 (23.8)	Not tested	
Anti-cardiolipin IgG	16 (76.2)	Not tested	
Anti-cardiolipin IgM	7 (33.3)	Not tested	
Lupus anticoagulant	13 (61.9)	Not tested	
Triple positive	4 (19.0)	Not tested	
Anticoagulant use, *n* (%)			
DOAC	4 (19.0)	0 (0.0)	0.113
VKA	9 (42.9)	0 (0.0)	0.002
LMWH	0 (0.0)	0 (0.0)	
None	8 (38.1)	17 (100.0)	<0.001
Antiplatelet therapy, *n* (%)	4 (19.0)	0 (0.0)	0.113

Abbreviations: APS, antiphospholipid syndrome; BMI, body mass index; DOAC, direct oral anticoagulant; LMWH, low-molecular weight heparin; NA, not applicable; SD, standard deviation; SLE, systemic lupus erythematosus; VKA, vitamin K antagonist; β2GP1, β2 glycoprotein 1.

a APS diagnosis according to the 2006 Sydney classification criteria. 1

## Results

Twenty-one APS patients and 17 control participants were included ( Table 1 ), of which 15 APS patients and 16 control participants underwent shotgun sequencing for identifying microbial proteins with mimotopes (anonymized data available in a repository).

### Selected Microbial Proteins of Interest

Fourteen B and T cell-mimicking microbial protein combinations were identified using the in silico pipeline. Additional quality checks disqualified six protein combinations due to the position of the B cell mimotope in the predicted tertiary protein structure, which suggested poor availability for antibody binding. One combination was disqualified due to an unknown T cell protein. Seven protein combinations were eligible for in vitro testing. The seven B-cell mimicking proteins were submitted for production, of which six were successfully recombinantly expressed.

**Fig. 2:**
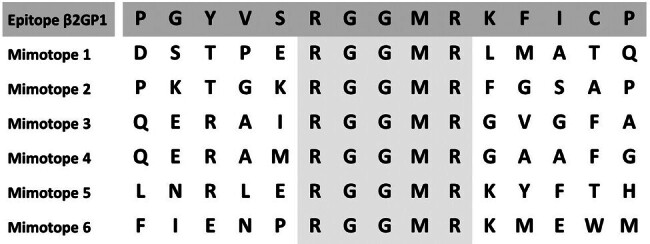
Epitope alignments of B cell proteins. The top row shows the “RGGMR” B cell epitope of β2GP1 and the surrounding five amino acids. The following rows show amino acids surrounding “RGGMR” in the selected mimotopes.

An overview of the six identified microbial protein combinations and their characteristics is summarized in Table 2 , and epitope alignment is displayed in Fig. 2 . T cell mimotopes varied in alignment length from 6 to 12 amino acids. Alanine-tRNA ligase identified in *Alistipes putredinis* was most prevalent in APS patients ( *n* = 9). Three identified microbes belonged to the Roseburia genus. The one gut microbial protein of which a cross-immunizing role in APS pathophysiology has previously been reported, DNMT from *R. intestinalis* , 6 was also identified by our in silico approach as a potential mimotope-containing microbe. The microbial proteins with B-cell mimotopes were tested for immunoglobulin binding in the ELISA and were renamed protein 1 to 6 for convenience.

**Table 2:** Identified intestinal microbial proteins of interest

Genus/species	B-cell protein of interest	NCBI accession number: B cell protein	B cell epitope a	Rename the B cell protein	T cell protein of interest	T cell mimotope	T cell epitope	Alignment length T cell mimotope	APS patients ( *n* ) b	Controls ( *n* ) b
*Alistipes putredinis*	Alanine-tRNA ligase	WP_227088546.1	RGGMR	Protein 1	Helix-hairpin-helix domain-containing protein	LNGADSA	NTGFYLNGADSAKCT	7	9	11
*Parabacteroides distasonis*	NPCBM/NEW2 domain-containing protein	WP_154397729.1	RGGMR	Protein 2	Sugar-binding domain-containing protein	TGFYLN	NTGFYLNGADSAKCT	6	6	1
*Roseburia intestinalis*	2-iminoacetate synthase ThiH	WP_154397729.1	RGGMR	Protein 3	YceG family protein	VKMGTVAYHAER	PVKKATVVYQGERV	12	7	5
*Eggerthella*	2-iminoacetate synthase ThiH	WP_009608642.1	RGGMR	Protein 4	SGNH/GDSL hydrolase family protein	TVYYQGER	PVKKATVVYQGERV	8	4	0
*Roseburia amylophila*	YjjG family noncanonical pyrimidine nucleotidase	WP_227709576.1	RGGMR	Protein 5	Hypothetical protein	TVEYQGEKV	PVKKATVVYQGERV	9	4	1
*Roseburia intestinalis*	DNA methyltransferase 6	WP_118597735.1	RGGMR	Protein 6	Hypothetical protein 6	FFCINKE	KVSFFCKNKEKKCSY	7	5	10

a All six identified B cell proteins contained mimotopes with 100% similarity to the B cell epitope.

b Number of APS patients and controls with the specific protein combination present.

### Cross-Reactivity to Microbial Proteins

#### IgG Reactivity in APS Patients and Control Participants

**Fig. 3:**
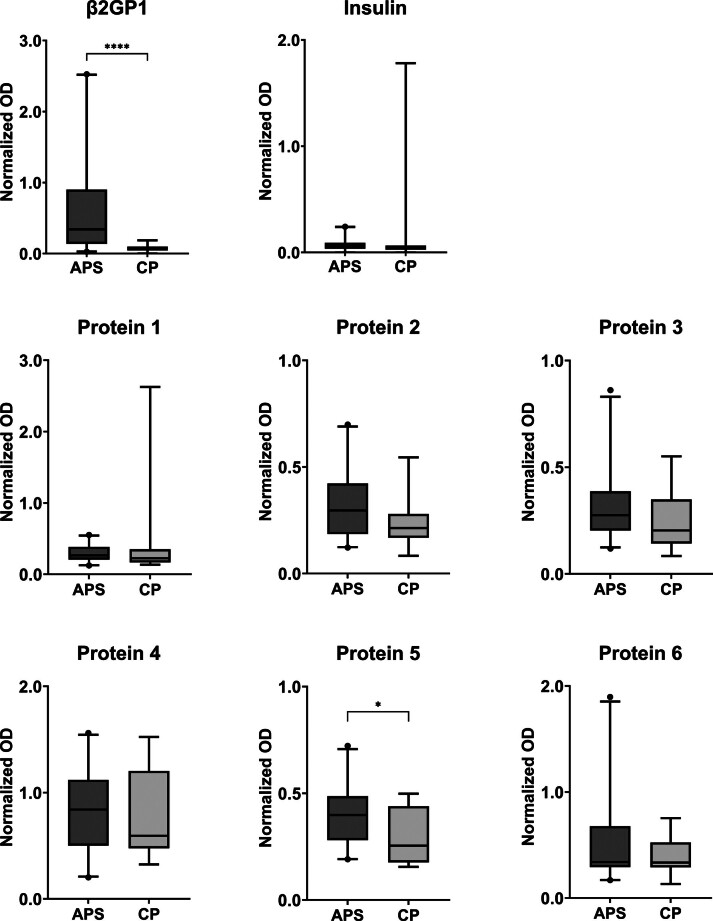
IgG reactivity of APS patients (APS, *n* = 21) and control participants (CP, *n* = 17) to β2 glycoprotein 1 (β2GP1), insulin, and six microbial proteins of interest. β2GP1 served as the positive control, and insulin as the negative control. Microbial proteins of interest. Data is expressed as median with interquartile range and analyzed using a Mann–Whitney U test. **** *p* < 0.0001, * *p* < 0.05. OD, optical density.

The reactivity of plasma IgG of APS patients and control participants toward candidate microbial proteins was tested. IgG reactivity to the positive control β2GP1 was increased in APS patients compared to control participants, *p* < 0.0001 ( Fig. 3 ). IgG reactivity to five microbial proteins of interest did not differ between APS patients and control participants ( Fig. 3 ). IgG reactivity to one protein of interest was increased in APS patients compared to control participants: protein 5, YjjG family noncanonical pyrimidine nucleotidase expressed by *Roseburia amylophila* , *p* = 0.035 ( Fig. 3 ).

To further test anti-β2GP1 reactivity to protein 5, we tested protein 5 with the β2GP1-specific mouse monoclonal antibody 3B7. 18 Since cross-reactivity of protein 6 was published before, we also included this protein. 6 The monoclonal antibody showed increased reactivity to proteins 5 and 6 compared to insulin ( Supplementary Fig. S1A ), but reactivity was much lower than to β2GP1, the antigen for which this monoclonal was developed, which saturated at 0.03 µg/mL ( Supplementary Fig. S1B ).

#### Cross-Reactivity to YjjG Family Noncanonical Pyrimidine Nucleotidase in APS Patients

**Fig. 4:**
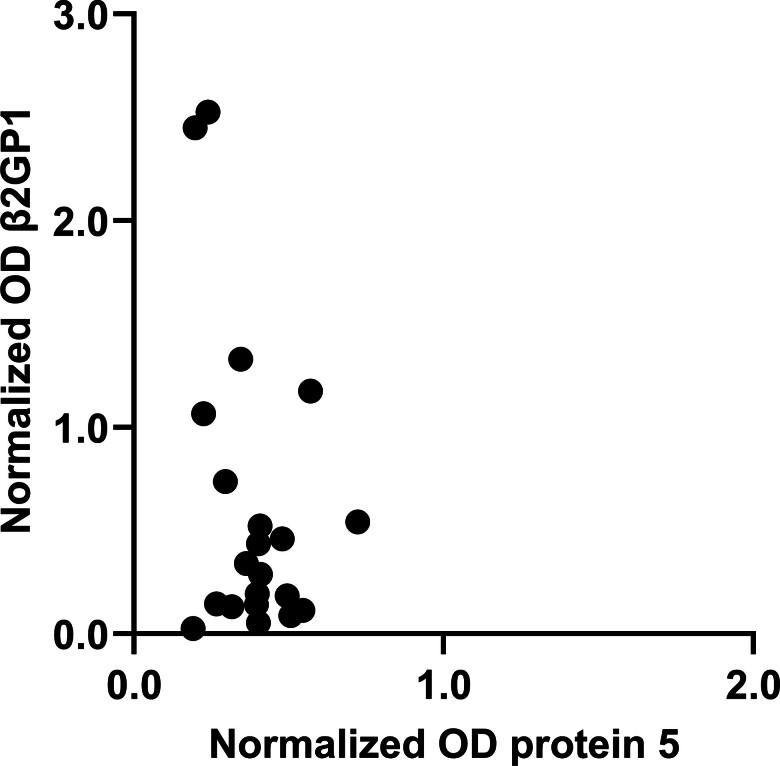
Correlation analysis of IgG reactivity to protein 5 and β2 glycoprotein 1 (β2GP1) in APS patients ( *n* = 21). Data is analyzed using Spearman's rank-order correlation, *r* = −0.155 and *p* = 0.503. OD, optical density.

The above results demonstrate that APS patients have higher IgG reactivity to YjjG family noncanonical pyrimidine nucleotidase from *R. amylophila* than control participants in our cohort. This may reflect either immunization to this gut microbial antigen or anti-β2GP1 IgG cross-reactivity with this mimotope. However, the absence of a correlation between anti-β2GP1 IgG reactivity and anti-YjjG family noncanonical pyrimidine nucleotidase IgG reactivity does not support mere cross-reactivity, *r* = −0.155, *p* = 0.503 ( Fig. 4 ).

#### IgM and IgA Reactivity in APS Patients and Control Participants

We tested the reactivity of plasma IgM and IgA of APS patients and control participants toward candidate microbial proteins. Both IgM and IgA reactivity to β2GP1 were significantly increased in APS patients compared to control participants, but neither IgM nor IgA reactivity to the candidate proteins was different in APS patients compared to control participants ( Supplementary Figs. S2 and S3 ).

## Discussion

Cross-immunization by molecular mimicry of intestinal microbial proteins might contribute to the formation of aPL in APS patients. In this study, we aimed to identify and assess intestinal microbial proteins with an epitope mimicking the B-cell epitope of β2GP1, the major autoantigen in APS. A purpose-built in silico pipeline was used to identify six gut microbes with β2GP1 epitope mimicking proteins in APS patients. We tested the B-cell mimotope-containing proteins in an in-house developed ELISA for immunoglobulin reactivity in APS patients and control participants. APS patients show increased IgG reactivity to YjjG family noncanonical pyrimidine nucleotidase expressed by *R. amylophila* compared to control participants. In line with this observation, an anti-β2GP1 monoclonal antibody also showed higher affinity to this protein than to a negative control. We found no difference in IgG reactivity to five in silico identified microbial proteins in APS patients compared to control participants.

*R. amylophila* is a recently discovered microbe, and its function in the intestinal microbiome is unknown. 21 The secondary outcomes comprising IgM and IgA reactivity to the microbial proteins showed no difference between APS patients and control participants. Some control participants also showed reactivity to some of the mimotopes, which could be expected as part of normal mucosal immunity to these gut commensals. Due to small subgroups, we did not perform subgroup analyses.

Increased IgG reactivity to DNMT of *R. intestinalis* was previously shown in APS patients. 6 In line with these findings, our bioinformatics pipeline identified this specific mimicking protein as well. Although we did not observe increased IgG reactivity to DNMT in APS patients, a β2GP1-specific monoclonal antibody did show binding to DNMT. Our results thus only partially align with the previous report. Since there is reactivity of DNMT with the monoclonal antibody, it seems to have cross-reactive properties in our study as well. It is notable that two intestinal microbes from the same family both express a protein with molecular mimicry to β2GP1. *R. intestinalis* is a gram-positive anaerobe that is curved and rod-shaped with a flagellum. It produces short-chain fatty acids and is known for its butyrate production. Together with another bacterium, it is the most abundant butyrate-producing bacterium in human feces. It is a common gut microbe, and the *R. intestinalis* cluster accounts for approximately 2.3% of the total human gut microbiome. 22 *R. amylophila* has recently been characterized as a Roseburia species and has not been studied elaborately yet. 21 Both species are not infectious species but reside in the human gut as commensals.

The identified enriched binding of one of the identified mimotopes in APS patients compared to controls is suggestive but not evidence of cross-immunization by this commensal. In the hypothesized pathophysiological role of gut commensals, that is, B-cells with affinity for the mimotope being stimulated and matured and eventually producing antibodies that are also reactive to the resembling β2GP1, APS patients would have both anti-mimotope and anti-β2GP1 reactivity. But given the underlying polyclonal B cell population, each clone with its own abundance and antibody-forming capacity, these reactivities, though both present, do not necessarily correlate. Work on posttranslationally modified antigens has, for instance, shown that cross-immunization can indeed occur in autoimmunity, but the strength of correlation between antibody titers against the separate antigens differs and is context dependent. 23 Moreover, mucosal immunity has been found to respond differently than the classical view of systemic humoral memory responses in the sense that the current bacterial load is an important determinant of IgA titers. 24 On the other hand, if the observed anti-mimotope reactivity reflected mere in vitro cross-reactivity to β2GP1, you would expect a clear correlation between the two antibody “titers.” Further analysis of the YjjG family noncanonical pyrimidine nucleotidase IgG reactivity showed no correlation between the level of IgG reactivity with this protein and β2GP1 in APS patients. Though not directly evidenced by our human participant study, this finding may thus argue against in vitro cross-reactivity as an explanation for the higher anti-YjjG family noncanonical pyrimidine nucleotidase titers in APS patients. A role of this microbe in APS pathophysiology through in vivo cross-immunization would, however, align with these data, where titers of antibodies directed at the mimotope and auto-epitope would not necessarily correlate.

This study is based on robust APS patient-derived intestinal microbiome data with the use of an in silico pipeline, complemented by ELISA reactivity testing. This cross-disciplinary approach allowed us to probe the entire intestinal metagenome for microbial proteins contributing to APS etiology. IgG, IgM, and IgA reactivity data showed relevant levels of variation, likely due to inherent heterogeneity in adaptive immune responses to these gut commensals. This, in combination with a relatively modest sample size, may have masked possible differences in reactivity to the other five mimotopes between APS patients and controls. Another potential source of variation is heterogeneity amongst APS patients in terms of clinical phenotype and types and titers of antibodies. Moreover, due to the cross-sectional nature of the study cohort, the pathophysiological stage of APS that the included patients were in, in terms of developing autoimmune responses, varies within the cohort, as these were not all recently diagnosed APS patients. We only included female patients with APS; our results may not be applicable to male patients with APS. Future directions for research can include testing the expression of the identified proteins by the gut microbes, testing the T cell mimotopes, and identifying the Roseburia genus in a larger group of patients with APS. While *R. intestinalis* is an established gram-positive, butyrate-producing commensal, *R. amylophila* has only recently been identified. 6 21

In conclusion, in search for cross-immunization by intestinal microbial proteins, we observed increased IgG reactivity to YjjG family noncanonical pyrimidine nucleotidase from *R. amylophila* in APS patients. Further research is needed to pinpoint the role of the intestinal microbiome in APS pathogenesis, which could be focused on the Roseburia genus.

## Notes

**What is Known About This Topic?**

- Patients with antiphospholipid syndrome (APS) lose tolerance to β2 glycoprotein 1 and form antibodies against this protein that cause thrombotic and obstetric events.
- Molecular mimicry by pathogens is a well-established phenomenon.

**What Does This Paper Add?**

- We hypothesize intestinal microbes contribute to loss of tolerance by expressing β2 glycoprotein 1 mimics.
- We identified intestinal microbial proteins to which anti-β2 glycoprotein 1 antibodies cross-react.
- These potential cross-immunizing proteins might be essential for autoantibody formation in APS patients.
