# Supplementary material for: Cross-Reactivity of Antiphospholipid Antibodies with Gut Commensal Proteins in Antiphospholipid Syndrome
Source: TH Open. 2026 Jun 3;10:a28685248. doi: 10.1055/a-2868-5248 (PMC13289573; doi:10.1055/a-2868-5248)
Supplement: Supplementary file 1 — Supplementary Material [file 10-1055-a-2868-5248_28885869.pdf]

Supplementary Materials and Methods

S2.4 *In-silico* pipeline for microbial protein selection

The goal of the *in-silico* pipeline was to identify gut microbial proteins with an epitope homologous to the B cell epitope of β2GP1 in APS patients. As a requirement a β2GP1 T cell epitope homologue should be present in the same metagenome-assembled genome, i.e., microbe, as both B and T cell responses against β2GP1 are involved in APS autoimmunity and thus both a β2GP1 B and a T cell mimotope are necessarily present in a microbe in order to trigger cross-immunization. The overall approach started with feces of APS patients to identify presence of homologous gut microbial proteins.

S2.4.1 Read trimming and contig assembly

Processing of the fecal sequencing data started with adapter trimming and quality control of the raw 150 bp paired-end reads using fastp (v0.20.1) with standard quality settings.[1] Trimmed reads were mapped in very-sensitive mode to the human genome (build GRCh37) with bowtie2 (v2.4.2), considering dovetail mates to be concordant. To ensure that the potentially discovered mimotopes are not human, SAMtools (v1.12) was used to convert SAM to BAM and afterwards used to select only unmapped paired-end reads with a primary alignment.[2] Selected non-human reads were sorted by name and converted back to forward and reverse fastq files with bedtools (v.2.30.0).[3] Reads were assembled per sample into contigs, using SPAdes (v.3.15.2) with option meta in only-assembler mode.[4] Assemblies from all samples were concatenated and contigs shorter than 2500 bp were removed with seqkit (v.0.16.0).[5] Contigs were deduplicated with dedupe from BBMap (v38.18) with a minimal identity cutoff of 95%.

S2.4.2 Read mapping, binning, and quality score

Since we were interested in bacteria that contained both a mimicry sequence of the T cell and B cell epitope, which are not necessarily on the same contig, contigs of the cleaned overall assembly were binned into larger metagenome-assembled genomes using MetaBat2 (v2.15), saving cluster membership of the contigs.[6] The depth of coverage for all samples, required for MetaBat2, was obtained with the MetaBat function 'jgi_summarize_bam_contig_depths' on the non-human reads that were mapped to the overall cleaned assembly using bowtie2 (v2.4.2), and thereafter converted to sorted bam files (SAMtools v1.12). A summary of bin completeness, strain heterogeneity, and contamination was obtained with CheckM (v.1.2.0), option lineage_wf.[7] To evaluate bin quality, we adapted a widely used quality score.[8-10] Since strain heterogeneity, indicated as the proportion of contamination that is likely from the same or similar strain, could be misassigned due to the concatenation of different sample-specific assemblies, we only considered the non-strain heterogeneity contamination of relevance. We therefore used equation 1:

$$QS_{no\ strain} = Completeness - 5 * \left(\frac{100 - strain\ heterogeneity}{100} * contamination\right) \quad (1)$$

S2.4.3 Protein prediction and mimotope identification

Prediction of protein-coding genes on the overall cleaned assembly was performed by Prodigal (v2.6.3) using the metagenomic mode.[11] The protein sequences in the protein translation file were converted to a blast-db protein database from the blast-2.12.0+ software

[12]. This database was queried for the T cell epitopes of interest: ‘NTGFYLNGADSAKCT’; ‘PVKKATVVYQGERV’; and ‘KVSFFCKNKEKKCSY’ using blastp (-task blastp-short, -evalue 200000) from the blast-2.12.0+ software.[12] Only ungapped alignments were considered for further analysis because the mimotopes should fit in the MHC II binding site, which fits around 15 specific adjacent amino acids. Since the B cell epitope ‘RGGMR’ was small (five amino acids), we only selected exact matches with this sequence using regular expressions (regex v2020.10.15, python 3.7.9) instead of blastp.

S2.4.5 Mimotope selection

Identified ungapped T and B cell mimotopes were assigned to a bin based on the contigs on which they were located. For further analysis, mimotopes on binned contigs were considered. All possible combinations of a T cell mimotope with a B cell mimotope on the same bin were then selected. Since the same (or very similar) protein can be located on different contigs (which could be present in different samples), but are in fact the same protein, we clustered the protein sequences of the proteins in which a T or B cell mimotope was located with the webserver of CD-HIT (v4.7) using default settings (on 13-07-2022).[13] Proteins for T cell mimotopes were clustered separately from the proteins for the B cell mimotope. T cell and B cell mimotope combinations were then assigned to a unique T&B cell protein cluster ID.

Full protein sequences of the protein in which the identified T and B cell mimotopes were identified were obtained from the total Prodigal output. In addition, the genomic location of the protein on the contigs was obtained and used to calculate the coverage (defined as ≥ 1 read) and mean read depth of those proteins for each sample.

The final selection was made by selecting T and B cell protein clusters that were present in at least four APS cases. Individual T and B cell mimotope combinations in these

clusters were considered to be present in a sample if both full protein sequences of the identified T and B cell mimotope were covered for at least 50% in this sample and if the combined average read depth of both protein sequences was at least 1. T and B cell mimotope combinations on a bin with $QS_{no\ strain}$ <50 or with a T cell alignment with a score ≤40 were excluded.

After these selections, protein-to-protein BLAST in the NIH protein database was used to identify the protein name and subsequent species. The protein with the lowest E-value was used to verify if the epitope sequence was present. B cell mimotope containing proteins were also checked for accessibility of antibody-binding using the tertiary structure modeled by Swiss-model.

Supplementary References

1. Chen S, Zhou Y, Chen Y, Gu J. fastp: an ultra-fast all-in-one FASTQ preprocessor. *Bioinformatics*. Sep 1 2018;34(17):i884–i890. doi:10.1093/bioinformatics/bty560
2. Danecek P, Bonfield JK, Liddle J, et al. Twelve years of SAMtools and BCFtools. *Gigascience*. Feb 16 2021;10(2)doi:10.1093/gigascience/giab008
3. Quinlan AR, Hall IM. BEDTools: a flexible suite of utilities for comparing genomic features. *Bioinformatics*. Mar 15 2010;26(6):841–2. doi:10.1093/bioinformatics/btq033
4. Nurk S, Meleshko D, Korobeynikov A, Pevzner PA. metaSPAdes: a new versatile metagenomic assembler. *Genome Res*. May 2017;27(5):824–834. doi:10.1101/gr.213959.116
5. Shen W, Le S, Li Y, Hu F. SeqKit: A Cross-Platform and Ultrafast Toolkit for FASTA/Q File Manipulation. *PLoS One*. 2016;11(10):e0163962. doi:10.1371/journal.pone.0163962
6. Kang DD, Li F, Kirton E, et al. MetaBAT 2: an adaptive binning algorithm for robust and efficient genome reconstruction from metagenome assemblies. *PeerJ*. 2019;7:e7359. doi:10.7717/peerj.7359
7. Parks DH, Imelfort M, Skennerton CT, Hugenholtz P, Tyson GW. CheckM: assessing the quality of microbial genomes recovered from isolates, single cells, and metagenomes. *Genome Res*. Jul 2015;25(7):1043–55. doi:10.1101/gr.186072.114
8. Almeida A, Mitchell AL, Boland M, et al. A new genomic blueprint of the human gut microbiota. *Nature*. Apr 2019;568(7753):499–504. doi:10.1038/s41586-019-0965-1
9. Bowers RM, Kyrpides NC, Stepanauskas R, et al. Minimum information about a single amplified genome (MISAG) and a metagenome-assembled genome (MIMAG) of bacteria and archaea. *Nat Biotechnol*. Aug 8 2017;35(8):725–731. doi:10.1038/nbt.3893
10. Parks DH, Rinke C, Chuvochina M, et al. Recovery of nearly 8,000 metagenome-assembled genomes substantially expands the tree of life. *Nat Microbiol*. Nov 2017;2(11):1533–1542. doi:10.1038/s41564-017-0012-7
11. Hyatt D, Chen GL, Locascio PF, Land ML, Larimer FW, Hauser LJ. Prodigal: prokaryotic gene recognition and translation initiation site identification. *BMC Bioinformatics*. Mar 8 2010;11:119. doi:10.1186/1471-2105-11-119
12. Camacho C, Coulouris G, Avagyan V, et al. BLAST+: architecture and applications. *BMC Bioinformatics*. Dec 15 2009;10:421. doi:10.1186/1471-2105-10-421
13. Fu L, Niu B, Zhu Z, Wu S, Li W. CD-HIT: accelerated for clustering the next-generation sequencing data. *Bioinformatics*. Dec 1 2012;28(23):3150–2. doi:10.1093/bioinformatics/bts565

Supplementary Figures

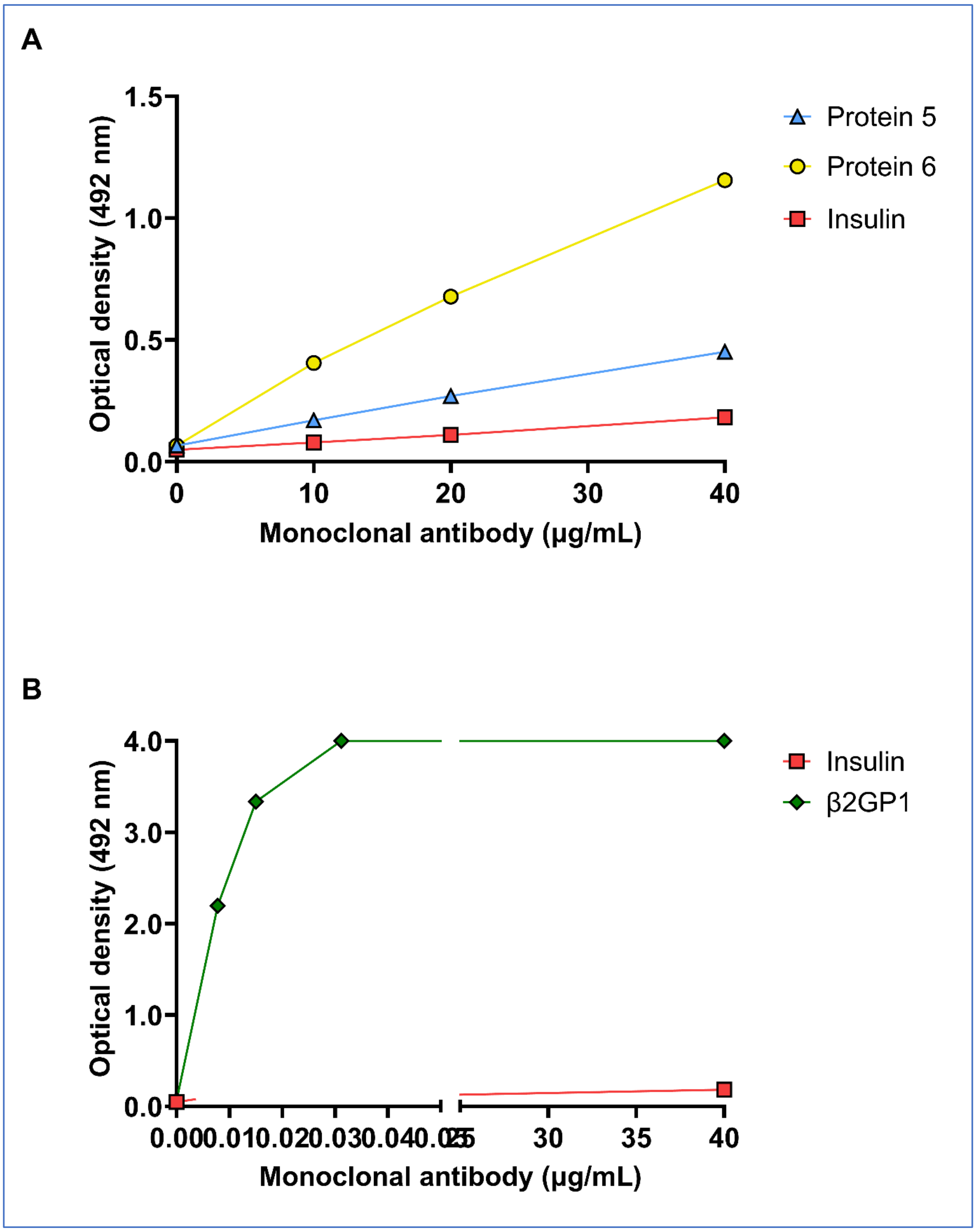

Figure S1: Specific β2 glycoprotein 1 mouse monoclonal antibody 3B7. A) Increasing dose of mouse monoclonal antibody in response to insulin, negative control, and two proteins of

interest. B) Increasing dose of mouse monoclonal antibody in response to β2GP1, positive control, and insulin, negative control. Data is expressed as a dose-response curve.

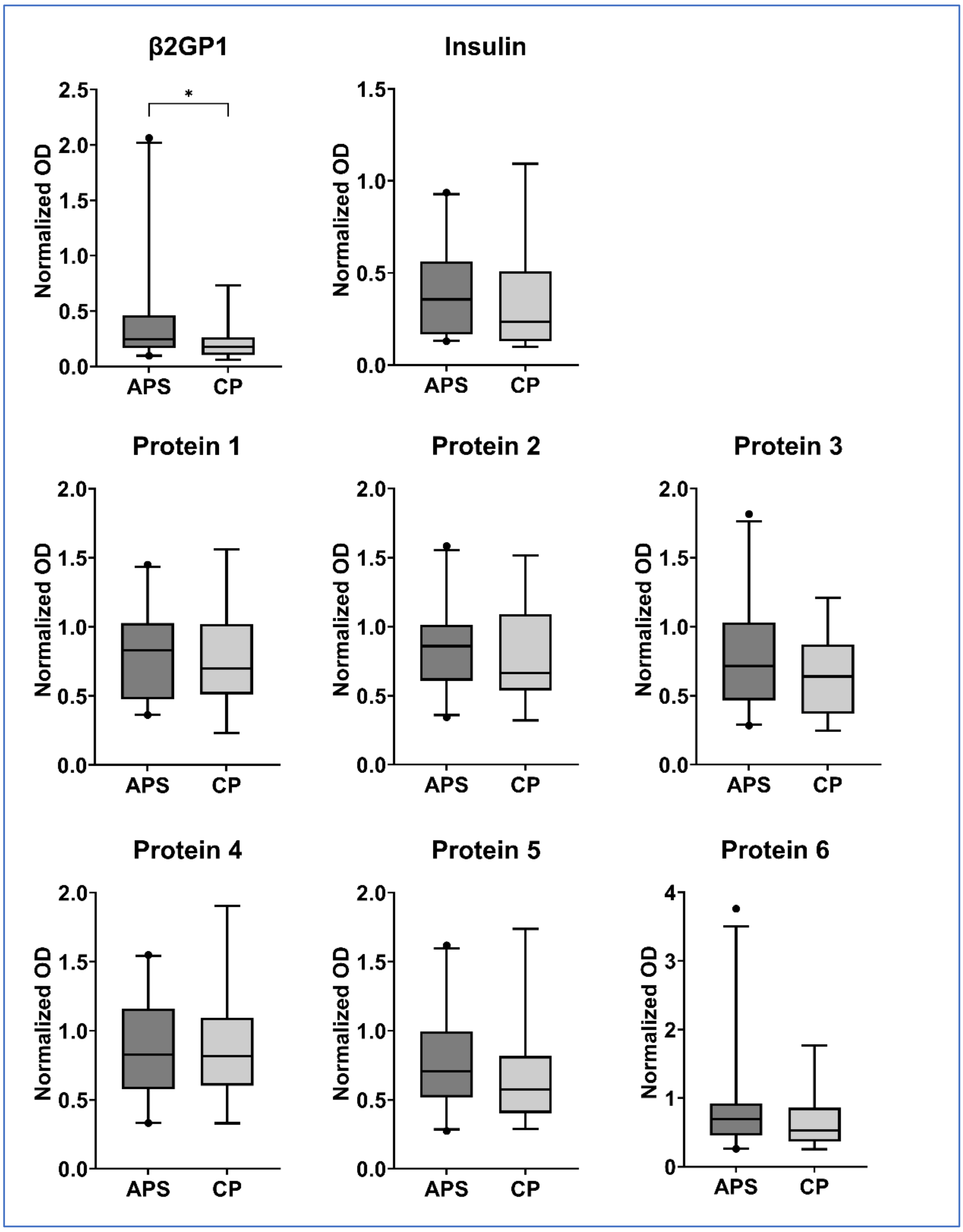

Figure S2: IgM reactivity of APS patients (APS, n=21) and control participants (CP, n=17) to β2 glycoprotein 1 (β2GP1), insulin, and six candidate microbial proteins of interest. β2GP1 served as the positive control, and insulin as the negative control. OD: optical density. Data is

expressed as median with interquartile range and analyzed using a Mann-Whitney U test. * p<0.05.

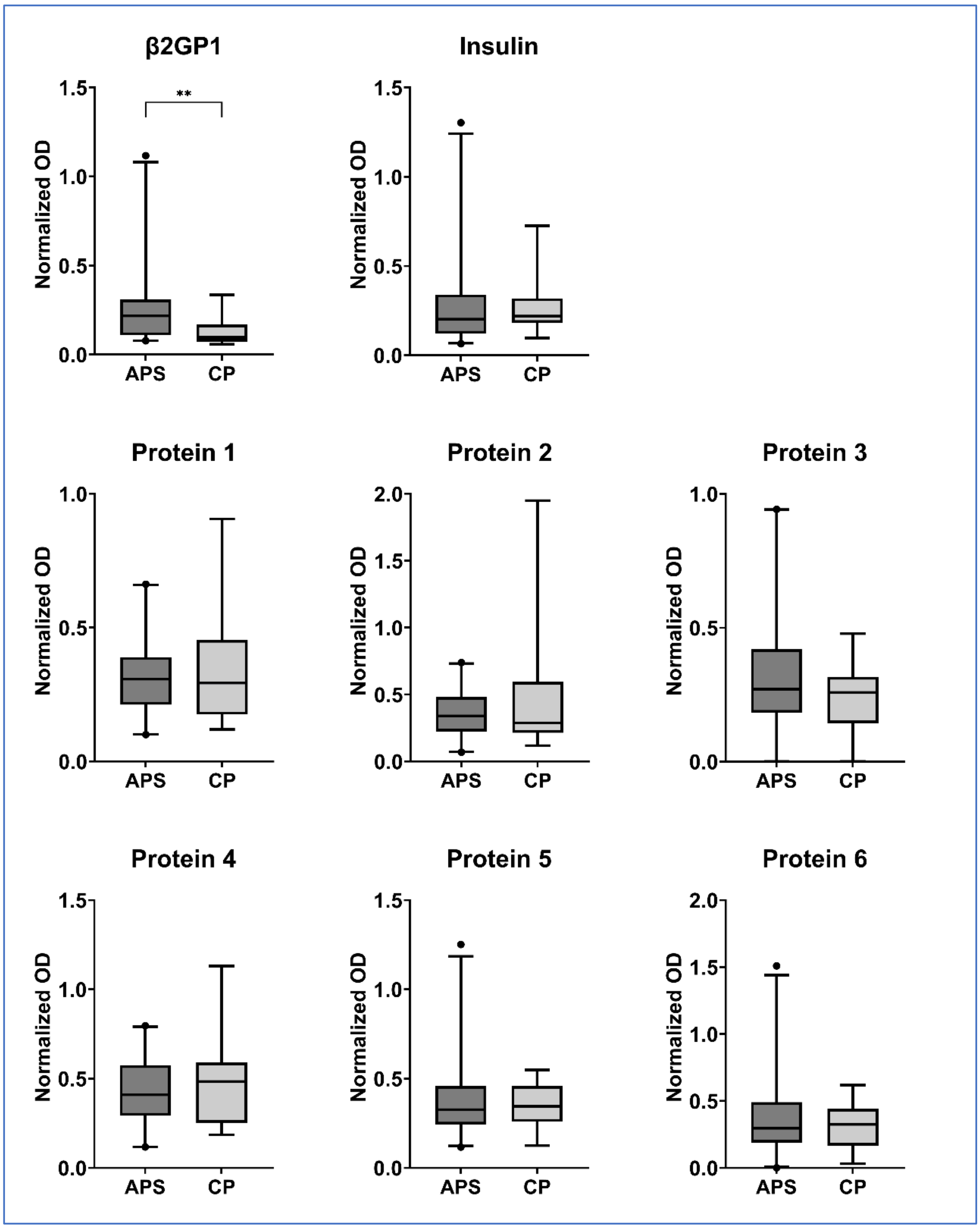

Figure S3: IgA reactivity of APS patients (APS, n=21) and control participants (CP, n=17) to β2 glycoprotein 1 (β2GP1), insulin, and six candidate microbial proteins of interest. β2GP1 is the positive control, and insulin is the negative control. OD: optical density. Data is expressed as median with interquartile range and analyzed using a Mann-Whitney U test. ** p<0.01.
